# Lanthanum associated abnormal liver function tests in two patients on dialysis: a case report

**DOI:** 10.1186/1752-1947-3-9321

**Published:** 2009-12-09

**Authors:** Girish Namagondlu, Norman Main, Lucy Yates, Joanne Mooney, Sangita Sathyamurthy, Indiver Daryanani, Alex Crowe, Tom Ledson, Anindya Banerjee

**Affiliations:** 1 Department of Nephrology, Arrowe Park University Teaching Hospital NHS Foundation Trust, UK; 2 Department of Nephrology, Countess of Chester NHS Foundation Trust, UK

## Abstract

Lanthanum (La) is a phosphate binder used in patients on dialysis in the UK. As it has only recently been in use, there are no long-term data about safety of this rare metal in human subjects with renal failure on renal replacement therapy. La has not been previously reported to cause any adverse reactions apart from nausea, sickness, dialysis graft occlusion and abdominal pain. We report here La induced abnormal liver function tests in a male and a female patient of 70 and 44 years old each, on peritoneal dialysis (PD) and haemodialysis (HD) respectively, the first report of such an adverse reaction to this agent.

## Introduction

Chronic kidney disease (CKD) is accompanied by disorders in bone and mineral metabolism, with effects on cardiovascular function and patient survival. In late stages of CKD, dietary modification is insufficient to control serum phosphate levels. Consequently, pharmacological therapy with an oral phosphate-binding agent is required to reduce the absorption of ingested phosphate.

A recent calcium and aluminium free phosphate binding agent approved for use in the UK is a metal based phosphate binder, Lanthanum carbonate [1]. La is a rare-earth trace metal that naturally occurs in monazite sand and coal and is a trivalent cation that acts as a calcium channel blocker [2,3].

We report here two cases of oral La induced abnormal liver function tests in patients on dialysis. To the best of our knowledge this is the first such report showing a possible association between oral La and abnormal liver function tests in patients on dialysis.

## Case Reports

### Case 1

A 70-year-old Caucasian man with end stage renal failure (ESRF) secondary to hypertensive nephrosclerosis on PD for one year presented with a one-day history of lower abdominal pain in November 2007. There was no history of altered bowel habits or features of sepsis. There were no other co-morbidities or any history of alcohol abuse or use of recreational drugs. His dialysis adequacy was reported as a Kt/V of 2.2/week, blood pressure (BP) was well controlled at 135/75 mmHg and residual urine output was about 0.7 l/day.

On examination he looked clinically well. His abdomen was soft without any other physical signs of any note.

His medication included Allopurinol, Atorvastatin, Sodium Valproate, Aspirin, Alphacalcidol, Omeprazole, Irbesartan, laxatives and Aranesp. La was added as a first-line phosphate binder about 2 weeks prior to admission at 750 mg thrice daily, the recommended starting dose of this binder instead of Sevelamer which he was taking at 2.4 g thrice daily. The rest of his medication had remained unchanged for several years.

He had a normal full blood count (FBC), bilirubin 16 micromol/L (normal 3-17 μmol/L), alkaline phosphatase (ALP) 574 IU/L (normal 40-129 iu/L), alanine transaminase (ALT) 301 IU/L (normal 8-45 iu/L), gamma-glutamyl transferase (GGT) 185 IU/L (normal 8-50 iu/L). Ferritin was 236 microgram/L (normal 22-322 μg/L) and C-reactive protein (CRP) was 2 mg/L (normal 1-10 mg/L). Routine liver function tests four weeks previously were normal with bilirubin 9 mmol/L, ALP 90 iu/L, ALT 9 iu/L, GGT 7 iu/L. Peritoneal fluid appeared clear. Gram staining and peritoneal fluid cultures were negative. His clotting parameters were normal.

A liver virology screen including hepatitis A, B, C was negative. Epstein - Barr virus (EBV) and cytomegalovirus (CMV) serology were negative. An ultrasound scan of his liver was reported as normal liver parenchyma texture. There was no evidence of gall-bladder calculi. An abdominal X-ray revealed some evidence of faecal overload and the pain was felt related to constipation. Treatment with additional laxatives had good effect.

La was stopped and Sevelamer restarted. His LFTs started improving within a week and were back to normal within 4 weeks of stopping La and blood results from December 2007 showed a bilirubin of 1 mmol/L, ALP 87 iu/L, GGT 16 iu/L, ALT 21 iu/L. Table 1 summarizes his blood test results.

**Table 1:** Biochemical test results for Case 1 during December 2007 before and after Lanthanum was commenced and withdrawn (oral lanthanum started on 01.11.2007 and stopped on 10.11.07).

	Bilirubin Micromol/l	ALP IU/L	ALT IU/L	Gamma GT IU/L	Albumin g/L
**Normal**	05-21	40-125	0-40	10-50	36-52
**03.10. 07**	09	90	09	07	34
**08.11. 07**	16	574	301	185	37
**15.11.07**	15	420	74	109	33
**19.11.07**	16	284	29	67	33
**22.11.07**	13	205	21	51	29
**22.12.07**	01	87	12	16	24

ALP-Alkaline phosphatase, ALT-Alanine Transaminase, GammaGT-Gamma -glutamyl transpeptidase

### Case 2

A 44 year old patient on HD presented with fluid overload in February 2008. She was diagnosed to have coronary artery disease which needed stenting during this admission.

She had ESRF from Type 1 diabetes diagnosed in 1988 and other diabetes associated complications. She also had mild mitral regurgitation and a history of cholecystectomy from gallstones. She never had good diabetes control and before commencing on renal replacement treatment in 2007 via PD her average HbA1C were about 11.5%. As she continued to have problems with nephrotic range proteinuria from poorly controlled diabetes with consequent fluid overload, she was switched to HD in December 2007. Following commencement of HD her fluid balance improved, blood pressure medications were minimized and her diabetes control improved significantly with an HbA1C 9.5% in February 2008. She did not drink alcohol or abuse recreational drugs. Her admission Kt/V was 1.6; her BP was 140/80 mmHg and her residual urine output was 1.5 l/day. Interdialytic weight gains ranged from 2-3 kg at each session.

Her medication list included Sevelamar, Alfacalcidol, Irbesartan, Citalopram, Quinine Sulphate, Bisoprolol, Levemir, Novorapid, Lansoprazole and Aspirin. Her admission phosphates were elevated at over 2 mmol/L (normal 0.8-1.4 mmol/L), hence in place of Sevelamer, La was started in March 2008 at a dose of 750 mg thrice daily. Her LFTs before admission were normal (bilirubin 8 mmol/L, ALT 19 iu/L, ALP 97 iu/L and GGT 22 iu/L). Ferritin and CRP were respectively 236 microgram/L and 6 mg/L. By April 2008 her ALP was 602 iu/L, GGT 699 iu/L and ALT 75 iu/L; a Gastroenterology review was sought at this point and a subsequent liver ultrasound scan was reported as normal.

Later in April 2008 during a routine HD clinic at a satellite dialysis unit she complained of incessant itching. Routine bloods confirmed jaundice with bilirubin 49 mmol/L with further derangement of other liver enzymes (ALT 89 iu/L, ALP 1006 iu/L, GGT 748 iu/L). Liver virology screen was negative and EBV and CMV serology were negative. Clotting parameters were normal.

As La was the only new agent started this was stopped and Renagel restarted. By the first week of May 2008 her bilirubin May 2008 her bilirubin was 14 mmol/L, ALT 43 iu/L, ALP 665 iu/L and GGT 404 iu/L. Table 2 summarizes her blood test results.

**Table 2:** Biochemical test results for Case 2 during March/April 2008 before and after Lanthanum was commenced and withdrawn (oral lanthanum started on 23^rd ^March 2008 and stopped on 23^rd ^April 2008).

	Bilirubin Micromol/l	ALP IU/L	ALT IU/L	Gamma GT IU/L	Albumin g/L
**Normal**	05-21	40-125	0-40	10-50	36-52
**23.01.08**	07	115	38	22	37
**23.03.08**	08	125	35	45	36
**17.04.08**	49	1006	89	748	31
**01.05.08**	14	665	43	404	31
**05.06.08**	07	205	35	87	32

ALP-Alkaline phosphatase, ALT-Alanine Transaminase, GammaGT-Gamma -glutamyl transpeptidase

## Discussion

To the best of our knowledge this is the first report of abnormal LFTs' in association with La in patients receiving dialysis.

Abnormalities of liver function tests in patients on chronic dialysis can be related to a variety of causes ranging from viral infection, congestion from fluid overload and drugs. Indeed necropsy findings of the hepatobiliary system from 78 patients with ESRD maintained on HD have shown 90% exhibited some form of an abnormality, such as congestion complicated by fibrosis, fatty metamorphosis, triaditis, hemosiderosis, and cystic changes along with chronic active hepatitis; almost 22% showed cholelithiasis [4]. Reports of electron microscopy examination of hepatic tissue mention marked proliferation of smooth endoplasmic reticulum in addition to alteration of mitochondria and rough endoplasmic reticulum and an increase in cytoplasmic lipid droplets [5].

The reported side effects in patients given La compared to patients given placebo for 4-6 weeks were nausea, vomiting, dialysis graft occlusion, and abdominal pain [6]. It is thought that as La transits through the liver via a trans-cellular pathway this organ is probably well protected against damage. In the liver La is mainly found in the lysosomes and biliary canaliculi. Although in clinical trials there was no difference in LFTs' between patients receiving La versus standard therapy [7], La does however accumulate in bone, gastrointestinal tract and liver of people and animals given this drug, particularly in CKD; in animal models of CKD this is from increased absorption [8], and in humans both the extent and the rate of absorption is greater than in individuals with normal kidney function [9]. This persistent accumulation and poor clearance of La once it enters tissues indicates that La has a long biological half-life with the potential to act as a cumulative toxin. Organ burden cannot be monitored by blood La levels [10]. Previous studies have shown trivalent cations to be causal in membrane rigidification processes [11] with changes in enzyme activity in chicken livers even at exceedingly low concentrations [12].

Neither of our cases demonstrated hepatic synthetic functional derangement as clotting parameters was within normal limits. Ferritin levels in both patients were within acceptable range and there was no evidence of an intercurrent sepsis to explain an abnormality in the LFTs. Both cases demonstrated biochemical cholestasis along with hepatocyte injury which promptly resolved on La withdrawal. This is particularly significant as both our cases were on the minimum recommended dose of this binder, the maximum dose recommended in patients with poor phosphate control being 4.5 g daily.

Average urine output and fluid balance were reasonable for both cases, hence we do not suspect fluid overload with liver congestion to be the cause of abnormal LFTs. Even if congestion or poor diabetes control were to be responsible for abnormal LFTs in Case 2, the prompt reversal of her jaundice once La was stopped indicates an association with this agent.

All renal failure patients are on a multitude of medications and our patients were not any different. Although some of these such as Sodium Valproate, Atorvastatin or even Omeprazole as in Case 1 and Lansoprazole in Case 2 could contribute to a biochemical picture of abnormal LFTs, the time course of events in our cases would seem to suggest otherwise, particularly as these patients had been on these agents for a number of years. La was the only new change to their prescription within about 2 weeks prior to presentation.

Re-challenging with La was felt unnecessary as alternative phosphate binding agents were available. As Case 2 was jaundiced this action may have had potentiated the hepato-toxic effect of La.

We did not have liver biopsy confirmation of lanthanum deposition or serum lanthanum levels and this is a potential shortcoming of our report. Serum La levels are not available easily; liver biopsy would have helped however the patients improved following withdrawal of La thus obviating a need for this invasive procedure. Additionally, both our patients had significant residual urine output in addition to adequate dialysis clearances. One can therefore speculate cumulative accumulation of La over only a few weeks of La use subsequently leading to La related hepatotoxicity is less likely in such a setting and that the abnormalities described could well be explained by chance alone; however 2 cases with a similar time scale of events should at least indicate that such an association is possible, whether from drug hepatotoxicity or an idiosyncratic reaction.

## Conclusion

The time course of events in this report seems to suggest a pharmacological hepatotoxic/cholestatic effect in association with La in 2 patients on dialysis; this paper however does not conclusively demonstrate La toxicity in patients on dialysis. Physicians should at least be alert to this feature of La as a possible complication. We suspect other long-term side-effects of La in human subjects on dialysis also require further investigation, particularly in view of its pharmacokinetics in CKD.

Recommendations and guidelines about La and the dosage it should be used at, can only then become clearer

## Consent

Written informed consent was obtained from the patient for publication of this case report. A copy of the written consent is available for review by the Editor-in-Chief of this journal.

## Competing interests

The authors declare that they have no competing interests.

## Authors' contributions

AB and AC conceived of the study; GN and NM drafted the manuscript. LY, JM, SS, ID and TL participated in its design and carried out the coordination. All authors read and approved the final manuscript.
